# VISUAL PROCESSING SPEEDS DIFFERENTIATE OUTCOMES OF DRIVING EVALUATIONS OF CONTROLS AND MEDICALLY AT RISK DRIVERS

**DOI:** 10.1093/geroni/igac059.2282

**Published:** 2022-12-20

**Authors:** Anne Dickerson, Victoria Penna

**Affiliations:** East Carolina University, Greenville, North Carolina, United States; East Carolina University, Greenville, North Carolina, United States

## Abstract

Visual processing speed is considered a critical factor for determining driving capacity in older adults. The specific research questions were: 1) is there a statistically significant difference in performance time between the medically at-risk (n=35) and controls (N=242), 2) does the type of medical condition (e.g., neurological, cognition, complex medical conditions) differentiate performance, and 3) can visual processing speed differentiate between fit and unfit drivers. A cross sectional quasi-experimental design was used to compare the visual processing reaction times between at-risk adult drivers and healthy controls. Participants were medically-at-risk drivers referred for a comprehensive driving evaluation to determine their fitness to drive. The Vision Coach™ "Full-Field-60" task was used to collect reaction times which required participants to tap 60 randomly illuminated red dots. One practice trial was followed by three testing trials that were averaged together. At-risk participants were divided into three diagnostic categories. Fitness-to-drive outcomes were pass, fail, or restrictions. A propensity score method based on age and gender was used to account for the difference in sample sizes by weighting the participants from the two studies for a fair comparison between the two groups. Independent t-tests found a significant difference t(275)=-6.42, p=< 0.001 in trial times between healthy controls (M=53 + 10.82) and medically-at-risk adults (M=72 + 17.04). No significant difference was found between the diagnostic groups (p=0.141), but the Vision Coach™ differentiated between those who "passed" and those who "failed" a driving evaluation (F(2,32)=8.28, p=0.001.

